# Remdesivir-induced severe hypoglycemia in an elderly man without diabetes: a case report

**DOI:** 10.1186/s40780-024-00406-1

**Published:** 2025-01-27

**Authors:** Yasunori Nagano, Hisae Aoki, Juri David, Naoko Iwahashi Kondo, Makimi Aizawa, Toshiyuki Sumita, Yuki Hamada, Yuki Yamamoto, Kaoru Yamada

**Affiliations:** 1 Department of Pharmacy, Sanraku Hospital, 2-5 Kandasurugadai, Chiyoda-ku, Tokyo, 1018326 Japan; 2 Department of Surgery, Sanraku Hospital, 2-5 Kandasurugadai, Chiyoda-ku, Tokyo, 1018326 Japan; 3 Department of Internal Medicine, Sanraku Hospital, 2-5 Kandasurugadai, Chiyoda-ku, Tokyo, 1018326 Japan

**Keywords:** Remdesivir, Hypoglycemia, COVID-19, Diabetes mellitus

## Abstract

**Background:**

Remdesivir is recommended to treat hospitalized patients with coronavirus disease 2019 (COVID-19). Remdesivir is known to affect glucose metabolism in individuals with and without diabetes. However, little is known about the possibility of hypoglycemia associated with remdesivir. Our case is the first report demonstrating the development of severe hypoglycemia following remdesivir treatment in an elderly man without diabetes.

**Case presentation:**

A 73-year-old man developed COVID-19 following surgery for sigmoid volvulus. The patient’s medical history included surgery for posterior correction of scoliosis, Chiari malformation type I, and syringomyelia. There was no history of diabetes mellitus. The patient was started on remdesivir (200 mg on day 1, followed by 100 mg intravenously daily until day 9). On day 7, following remdesivir administration, the patient developed severe hypoglycemia with a blood glucose (BG) level of 25 mg/dL. On day 8 and 9 he repeatedly developed severe hypoglycemia following administration of remdesivir. Considering the timing of administration, the patient’s hypoglycemia could have been caused by remdesivir. Therefore, his treatment with remdesivir was discontinued. After discontinuation, his BG levels normalized. The Naranjo algorithm, a tool for evaluating the causality of adverse drug reactions, classified the event as “Probable” (6 points).

**Conclusions:**

Remdesivir may have caused hypoglycemia in this case. Health care professionals should be aware of its potential effects on glucose metabolism and the risk of hypoglycemia when treating patients with remdesivir.

**Supplementary Information:**

The online version contains supplementary material available at 10.1186/s40780-024-00406-1.

## Background

Remdesivir is a broad-spectrum nucleotide analogue prodrug that inhibits viral RNA-dependent RNA polymerase, thereby preventing viral replication and transcription of severe acute respiratory syndrome coronavirus 2(SARS-CoV-2) [1, 2]. Remdesivir has been demonstrated in clinical trials to reduce the median recovery period, reportedly to 10 days compared to 15 days in the placebo group [3]. With remdesivir now widely used in the treatment of coronavirus disease 2019 (COVID-19), numerous studies attest to its overall safety profile [4, 5]. Remdesivir is recommended for the treatment of COVID-19 in both hospitalized and non-hospitalized patients, according to guidelines from the Japanese Association for Infectious Diseases and the Ministry of Health, Labour, and Welfare of Japan [6, 7].

Hypoglycemia is rare in individuals without diabetes mellitus. However, various medications, serious illnesses, endocrine deficiencies, and non-islet cell malignancies are known to cause non-diabetic hypoglycemia. Severe hypoglycemia may cause fainting or seizures and can be life-threatening; therefore, immediate medical intervention is required for severe hypoglycemia [8]. Although hypoglycemia is a well-known adverse effect of antidiabetic agents, drug-induced hypoglycemia may occasionally occur during treatment with medications commonly used in everyday clinical practice, including NSAIDs, analgesics, antibacterials, antimalarials, antiarrhythmics, antidepressants, and other miscellaneous agents [9]. Therefore, it is imperative to conduct a thorough investigation into the underlying causes of hypoglycemia in patients without diabetes, paying special attention to any drugs that may be the contributing factors [10].

While clinically effective, remdesivir has been associated with hyperglycemia, characterized by elevated blood glucose (BG) levels during and after treatment. Previous studies have confirmed this association in COVID-19 patients [11, 12].

We present the first documented case of remdesivir-induced hypoglycemia in an elderly man without diabetes. Information on remdesivir-related hypoglycemia is limited, and our literature review revealed no prior reports of severe hypoglycemia in non-diabetic elderly patients not receiving hypoglycemic medications.

### Case presentation

A 73-year-old Japanese man underwent surgery to relieve a sigmoid volvulus. His past medical history included Chiari malformation type I and syringomyelia, both with onsets unknown, and had underwent surgery for posterior correction of scoliosis 10 years prior to presentation. Due to syringomyelia, the patient had limb contractures, pressure ulcers, dysphagia, urinary retention, and inability to expel sputum.

Postoperatively, the patient developed dysphagia which resulted in gastrostomy; his respiratory failure worsened and eventually required orotracheal intubation, which once was temporarily extubated but re-intubated due to excessive sputum production. The patient required ongoing management for chronic pressure ulcers. He had no history of diabetes, with fasting BG levels over the past few months ranging from 73 to 105 mg/dL, and he reported no known allergies nor adverse drug reactions.

On day 157 of surgery for sigmoid volvulus, the patient developed a fever and tested positive for SARS-CoV-2 antigen. He was diagnosed with a mild case of COVID-19 since he showed no signs of hypoxemia, nor his computed tomography (CT) scan showed any abnormalities. His vaccination history against COVID-19 was unknown.

**Fig. 1:**
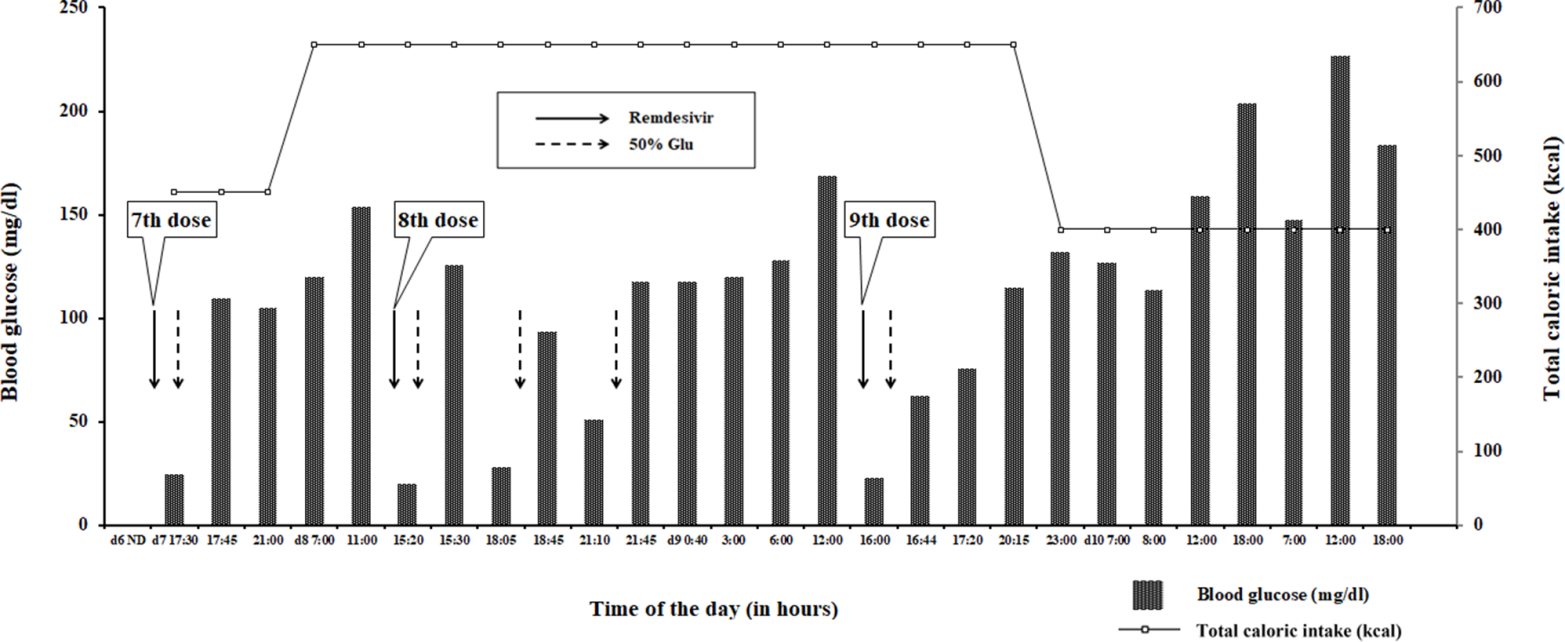
Details the relationship between the administration of remdesivir and the patient’s blood glucose levels and total daily caloric intake

The patient received an initial dose of 200 mg of remdesivir intravenous (IV), followed by a maintenance dose of 100 mg IV for 8 days, as he was considered to be at high risk of developing severe COVID-19. While administration of remdesivir, the patient’s other medications were discontinued. The discontinued medications included Clostridium butyricum MIYAIRI 588, L-carbocisteine, dimeticon, lemborexant, distigmine bromide, levofloxacin hydrate, droxidopa, silodosin, famotidine, sodium ferrous citrate. Table 1 shows changes in clinical laboratory data during remdesivir treatment. As for nutritional administration, previous to COVID-19 infection, the patient was enterically administered 1800 kcal (amount of glucose: 183.6 g) per day, which was switched to a peripheral parental infusion between 400 and 650 kcal (amount of glucose: 112–162 g) per day. Figure 1 shows a detailed relationship between remdesivir administration, the patient’s BG levels, and total daily caloric intake.

**Table 1:** Clinical laboratory data during the treatment of remdesivir

Clinical parameter (units)	Before initiation of remdesivir (day − 3)	At initiation of remdesivir (day 1)	At initiation of remdesivir (day 3)	During treatment with remdesivir (day 7)
WBC (count/L)	10.5 × 10^9^	11.3 × 10^9^	18.0 × 10^9^	20.9 × 10^9^
RBC (count/L)	368 × 10^10^	392 × 10^10^	370 × 10^10^	392 × 10^10^
Hb (g/L)	81	89	82	89
Hct (%)	27.5	29.1	27.5	27.8
Plt (count/L)	517 × 10^9^	479 × 10^9^	486 × 10^9^	479 × 10^9^
CRP (mg/L)	6.70	10.5	18.7	19.6
Alb (g/L)	23	25	21	18
AST (U/L)	16	23	27	35
ALT(U/L)	13	17	15	27
Cr (mg/dL)	0.30	0.35	0.31	0.24
BG (mg/dL)	79	131	115	25

Alb albumin, ALT alanine transaminase, AST aspartate transaminase, Cr creatinine, CRP C-reactive protein, BG blood glucose, Hb hemoglobin, Hct hematocrit, Plt platelets, RBC red blood cell, WBC white blood cell

On day 7, five hours after remdesivir administration, the patient developed hypoglycemia, with the lowest recorded BG levels being 25 mg/dL, along with presenting impaired consciousness and tachycardia. 50% glucose solution (50% Glu) was immediately administered IV; within 15 min, he regained consciousness, and his BG levels returned to normal. His head CT scan did not reveal any acute lesions.

On day 8, one hour and 20 min after remdesivir administration, his blood test again revealed hypoglycemia. However, there was no impairment of consciousness. 50% Glu was immediately administered IV, and within 10 min, his BG levels returned to normal. Two additional episodes of hypoglycemia followed on the same day. Each time, administration of 50% Glu restored his BG levels to normal.

On day 9, two hours after remdesivir administration, the patient again developed symptoms of hypoglycemia. Likewise, his BG levels returned to normal within 10 min following 50% Glu administration IV.

After assessing the patient’s medical history and clinical symptoms, we determined that remdesivir was likely the cause of hypoglycemia; consequently, we discontinued the planned administration on day 10. Subsequently, no further hypoglycemic episodes were observed.

The patient developed pneumonia and received piperacillin/tazobactam treatment. However, the patient’s condition worsened, eventually resulting in death.

## Discussion and conclusions

We present the case of a non-diabetic patient with COVID-19 infection who developed hypoglycemia 7 days after initiating remdesivir treatment that recurrently occurred upon its re-administration. This case report provides novel insights into the safety profile of remdesivir, with implications for patient care and BG monitoring protocols.

Hypoglycemia is an uncommon clinical condition in patients without diabetes, manifesting in the fasting or postprandial state, with some individuals experiencing both fasting and postprandial hypoglycemia [13]. Severe hypoglycemia can cause loss of consciousness or experience seizures. Severe hypoglycemia requires immediate treatment as it may be life-threatening. Consequently, it is crucial to investigate the underlying etiology of hypoglycemia in patients without diabetes comprehensively, with particular attention to potentially causative medications. The fulfillment of Whipple’s triad supports the presence of pathological rather than physiological hypoglycemia [14]. Whipple’s triad includes hypoglycemia with BG levels less than 55 mg/dL, the onset of autonomic or neuroglycopenic symptoms such as palpitations, sweating, anxiety, hunger, or confusion, and the remission of symptoms upon the administration of glucose. In this case, two of Whipple’s triad, symptomatic hypoglycemia (BG < 55 mg/dL) and its resolution after glucose administration, were observed ahead of medical evaluation.

Malnutrition can cause hypoglycemia. However, in this clinical scenario, an increase in BG levels was observed after discontinuing remdesivir therapy on day 10 despite no changes in caloric intake, making malnutrition unlikely a contributor. During treatment with remdesivir, the patient’s other medications asides IV fluids were discontinued, making in vivo accumulation unlikely. The IV fluids were never changed in content or amount throughout the administration and after discontinuation of remdesivir. Hence no pharmacological agent other than remdesivir is considered to be the potential etiological factor for these hypoglycemic episodes. Previous clinical trials have identified hypoglycemia (< 70 mg/dL) in approximately 1% of COVID-19 patients [15]. A retrospective study from Wuhan reported that around 10% of COVID-19 patients with Type 2 Diabetes Mellitus experienced at least one episode of hypoglycemia (< 70 mg/dL) [16]. It is unclear whether COVID-19 infection directly influences BG levels, so we cannot rule out the influence of COVID-19 infection in this case. However, there has been a report that COVID-19 infection does not cause hypoglycemia in the absence of severe co-morbidities or medications associated with hypoglycemic episodes [17]. Therefore, we suggest that COVID-19 infection is unlikely the cause of hypoglycemia in this case.

To rule out the possible existence of malignancies that can cause non-islet cell tumor hypoglycemia, the patient’s chest and abdominopelvic CT scans were taken, both revealing no abnormal findings. Adrenal insufficiency was excluded as his cortisol levels (27.7 µg/dL) and ACTH levels (85.8pg/mL) were normal.

It is possible that Chiari malformation type I and syringomyelia may have affected the patient’s BG. Rekate et al. reported four cases of patients with Chiari malformation who had intermittent hyperinsulinemic hypoglycemia, and proposed that changes in intracranial pressure may have caused hyperactivity in deformed vagus nerve in these patients, which could have affected their pancreas to release insulin leading to hypoglycemia [18]. Our patient, however, had no worsening symptoms of Chiari malformation type I nor syringomyelia following COVID-19 infection, and had never experienced hypoglycemia prior to receiving remdesivir during hospitalization. We therefore considered that these conditions were not the cause of the hypoglycemia in this case. It was also suggested that hypoglycemia due to sepsis could not be completely ruled out, as an increase in WBC was observed on day 7 of the administration of remdesivir, and the patient subsequently died. Besides, the Sequential Organ Failure Assessment (SOFA) score, a tool for assisting in the diagnosis of sepsis [19], scored the event as 0 points. Sepsis-induced hypoglycemia has been appreciated in human and animal models with depleted glycogen storage, impaired gluconeogenesis, and increased peripheral glucose utilization implicated as contributing factors [20, 21]. However, we considered that sepsis likely did not affect his BG, as no hypoglycemia was identified after discontinuation of remdesivir.

Unfortunately, we did not examine a 72-hour fasting test in the present patient. Additionally, we were unable to measure plasma insulin, C-peptide, and proinsulin levels at the onset in this patient, which are essential for identifying hypoglycemia, due to the unavailability of residual samples. Most notably, this hypoglycemic event manifested after the administration of remdesivir, and hypoglycemia recurred upon reintroducing remdesivir therapy. Furthermore, the resolution of hypoglycemic episodes was evident after discontinuation of remdesivir, and no other medications were administered during that period. The Naranjo algorithm, a validated tool for assessing the causality of adverse drug reactions [22], demonstrated a probable association (cumulative score: 6) between the hypoglycemic episodes and remdesivir administration. Table 2 shows the patient’s score for the Naranjo algorithm for adverse drug reaction causality assessment.

**Table 2:** The patient’s score for the Naranjo algorithm for adverse drug reaction causality assessment

S. No.	Question	Yes	No	Do not know	Score
1	Are there previous conclusive reports on this reaction?	+ 1	0	0	0
2	Did the adverse event appear after the suspected drug was administered?	+ 2	−1	0	2
3	Did the adverse reaction improve when the drug was discontinued or a specific antagonist was administered?	+ 1	0	0	1
4	Did the adverse reaction reappear when the drug was re-administered?	+ 2	–1	0	2
5	Are there alternative causes (other than the drug) that could solely have caused the reaction?	−1	+ 2	0	0
6	Did the reaction reappear when a placebo was given?	−1	+ 1	0	0
7	Was the drug detected in the blood (or other fluids) in a concentration known to be toxic?	+ 1	0	0	0
8	Was the reaction more severe when the dose was increased, or less severe when the dose was decreased?	+ 1	0	0	0
9	Did the patient have a similar reaction to the same or similar drugs in any previous exposure?	+ 1	0	0	0
10	Was the adverse event confirmed by objective evidence?	+ 1	0	0	1
		Total score			6

Total score categories are defined as follows: adverse drug reaction (ADR) is: definite ≥ 9; probable 5–8; possible 1–4; doubtful 0

The safety profile of remdesivir has been extensively evaluated since its emergence. However, there is limited peer-reviewed literature describing hypoglycemia as an adverse effect related to remdesivir administration.

In a review of related cases, we identified one case report that implicated remdesivir as a cause of hypoglycemia. André et al. reported a case of transient lactic acidosis, elevated transaminases, and hypoglycemia (BG levels unknown) after the second dose of remdesivir in a patient with acute kidney injury (AKI) [23]. To our knowledge, there are no case reports in the literature detailing the follow-up and time to resolution of remdesivir-induced hypoglycemia in patients without diabetes. Cases of hyperglycemia due to remdesivir have been reported, which show an inverse physiological response in this case. Kim W et al. reported the study of risk factors of hyperglycemia in hospitalized COVID-19 patients receiving remdesivir as Body mass index ≥ 23 kg/m2, proton pump inhibitor use, cholinergic medication use, and cardiovascular disease [11]. In our case study, these risk factors are not included. Remdesivir improved hyperglycemia, insulin resistance, fatty liver and endotoxaemia in mice fed a high-fat diet in Li, Y. N. et al.’s study [24]. In contrast, two randomized controlled trials (with Chinese patients and a multi-ethnic group) showed similar increases in BG levels in the remdesivir- and placebo-treated groups [2, 3]. Therefore, it is unclear whether the pathophysiological mechanism causing hyperglycemia is due to the disease itself, or drugs administered. Further evidence is needed to clarify the effect of remdesivir on glucose metabolism.

The precise mechanism underlying remdesivir-induced hypoglycemia remains elusive; on the other hand, we have developed a pharmacological hypothesis for the involvement of nucleoside analogues in triggering the hypoglycemic effect. The role of adenosine in glucose homeostasis may be attributed to its ability to regulate, through its membrane receptors, processes such as insulin secretion, glucose release and clearance, glycogenolysis, and glycogenesis [25, 26]. The molecular architecture of adenosine and its structural analogues has been documented to modulate the binding affinity and selectivity for distinct adenosine receptor subtypes [27]. Remdesivir is not an adenosine analogue per se; it functions as a prodrug of an adenosine nucleoside analogue designated GS-441,524, which exhibits structural similarity to adenosine [28]. We hypothesized that bioaccumulation of GS-441,524, a remdesivir metabolite with a chemical structure similar to adenosine, could affect glucose metabolism and insulin response, potentially causing hypoglycemia. In healthy subjects, a report shows that the concentration of GS-441,524 showed almost the same profile on Day 1 and Day 5 [29], and another report describes the trough concentration remained generally constant in COVID-19 patients with normal renal function [30].Remdesivir was associated with an increased risk of AKI in patients with COVID-19, according to pharmacovigilance analyses of the US FDA Adverse Event Reporting System database, and the mean time to onset of AKI was 4.91 days [31].Although the exact mechanism has not been elucidated, over a quarter of patients hospitalized with COVID-19 have been reported to develop AKI [32].In the present case, we could not identify a significant decrease in urine output during the study period, which would cause delayed GS-441,524 excretion. Moreover, there was no increase in serum creatinine (sCr) levels, a diagnostic criterion for AKI. However, sCr levels are kept low in elderly patients (with low muscle mass), making it easy to overlook impaired renal function [33].Hence, we hypothesized that the accumulation of GS-441,524 caused his hypoglycemia, assuming that the patient developed AKI due to COVID-19 infection and remdesivir administration.

To the best of our knowledge, remdesivir has not previously been associated with hypoglycemia in non-diabetic individuals. We posit that our present case report is the first to describe such a relationship. Healthcare professionals should be vigilant regarding the potential adverse effects of remdesivir- induced hypoglycemia. They should also carefully monitor hypoglycemia symptoms and BG levels during the follow-up period in patients treated with remdesivir. Studies examining the impact of remdesivir on glucose metabolism and BG regulation are limited, and further research is warranted to elucidate potential indirect effects on hypoglycemia during antiviral therapy.

At the time of publication, the patient had passed away, and written informed consent was obtained from the family. This case report from Sanraku Hospital did not require ethical approval under the Ethical Guidelines for Medical and Biological Research Involving Human Subjects.

## Electronic supplementary material

Below is the link to the electronic supplementary material.

Supplementary Material 1

## Data Availability

The datasets used and/or analyzed during the current study are available from the corresponding author on reasonable request.

## Abbreviations

COVID-19: Coronavirus disease 2019
SARS-CoV-2: Severe acute respiratory syndrome coronavirus 2
BG: Blood glucose
CT: Computed tomography
IV: Intravenous
50%Glu: 50% glucose solution
AKI: Acute kidney injury
SCr: Serum creatinine
