# Supplementary material for: Remdesivir-induced severe hypoglycemia in an elderly man without diabetes: a case report
Source: J Pharm Health Care Sci. 2025 Jan 27;11:5. doi: 10.1186/s40780-024-00406-1 (PMC11770970; doi:10.1186/s40780-024-00406-1)
Supplement: Supplementary file 1 — Supplementary Material 1 [file 40780_2024_406_MOESM1_ESM.docx]

Date: December 20, 2024

Dr Kenji Ikemura

Section Editor

Journal of Pharmaceutical Health Care and Sciences

Dear Dr Kenji Ikemura:

We wish to re-submit the manuscript titled “Remdesivir-Induced Severe Hypoglycemia in an elderly man without diabetes: A case report”. The manuscript ID is JPHC-D-24-00200.

We thank you and the reviewers for your thoughtful comments.

The manuscript has benefited immensely from the insightful suggestions.

I look forward to working with you and the reviewers to move this manuscript closer to publication in Journal of Pharmaceutical Health Care and Sciences.

The authors rechecked the manuscript and made necessary changes according to the reviewers’ comments and suggestions. The point-by-point responses to the comments are attached herewith. All comments are reflected in the attached response letter.

We hope that this revised manuscript meets your expectations.

Thank you for consideration, and I look forward to hearing from you soon.

Sincerely,

Yasunori Nagano, Pharmacist

Sanraku Hospital, 2-5 kandasurugadai, chiyoda-ku, Tokyo, Japan

E-mail: nagano8931@sanraku.or.jp

Tel: +81- 3-3292-3981

RESPONSE TO REVIEWER 1：

We wish to express our appreciation to the Reviewer for the insightful comments, which have helped us improve the paper.

General comment: *The revised manuscript and responses to comments have enabled the authors to revise the manuscript appropriately. However, please note the following point.*

Response: We thank the Reviewer for this pertinent comment.

Comment1: *In the manuscript (page 4) the unit of nutritional administration is kcal, whereas in Figure 1 it is calories. It is recommended that the authors correct the unit in Figure1 to exact units (kcal or kilocalories).*

Response: In accordance with the Reviewer's comment, we have changed the unit in Figure1 to exact units:

“Total caloric intake (calories) ”

to

“Total caloric intake (kcal)”

Thank you again for your comments on our paper. We trust that the revised manuscript is suitable for publication.

RESPONSE TO REVIEWER 2：

General comment: *The authors have adequately addressed the comments provided. I believe the manuscript has reached the level suitable for publication in JPHCS.*

Response: We thank the Reviewer for this pertinent comment.

Thank you again for your comments on our paper. We trust that the revised manuscript is suitable for publication.
